# From Solo to Collaborative: The Global Increase in Neuroscience Authors Over Two Decades

**DOI:** 10.1111/ejn.70092

**Published:** 2025-03-31

**Authors:** Mariella Segreti, Ann Paul, Pierpaolo Pani, Aldo Genovesio, Emiliano Brunamonti

**Affiliations:** ^1^ Department of Physiology and Pharmacology Sapienza University Rome Italy; ^2^ Behavioral Neuroscience PhD Program Sapienza University Rome Italy; ^3^ Department of Pharmaceutical Sciences University of Piemonte Orientale Novara Italy

**Keywords:** ANVUR, authorship increase, neuroscience

## Abstract

The increase in the number of authors per article is a well‐documented phenomenon across various academic disciplines. While prior studies have examined this trend in specific fields and countries, they in most cases did not compare the increase in the number of authors between countries. While it has previously shown that the number of authors in neuroscience publications has risen in the G10 countries, no study has yet addressed whether it reflects a global trend in the field. To address this gap, we quantified the global trend in the number of authors in neuroscience publications from 2001 to 2022. Our findings reveal a consistent increase in authorship across nearly all the countries examined. Italy ranks highest in terms of average authorship per article, while Ukraine ranks the lowest. On the other hand, China shows the largest increase in authorship over the years, followed by Norway and Egypt. South Korea is the only country showing a slight decreasing trend rather than growth. These results contribute to a better understanding of authorship patterns in neuroscience and can stimulate further investigations on the reasons behind such an increase in terms of socio‐economic factors, the need for collaborative efforts in some fields, or, on the negative side, the effect of utilitarian reasons to meet career evaluation criteria.

## Graphical abstract

This study quantified the global trend in neuroscience publications from 2001 to 2022, revealing a consistent increase in authorship. The findings highlight authorship patterns in different countries and suggest further investigation into socio‐economic and collaborative factors, addressing differences between countries and historical periods.

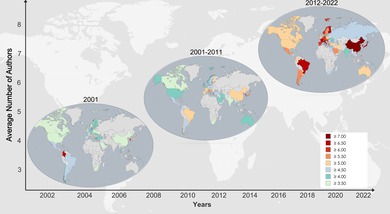

**Abbreviations**
ANVUR: Agency for the Evaluation of the University and Research System
ESI: Essential Science Indicators
G10: Group of Ten
GDP: gross domestic product
ICMJE: International Committee of Medical Journal Editors
PhD: doctor of philosophy
WoS: Web of Science

## 1 Introduction

The increase in the number of authors per paper has been shown across various academic disciplines (Khan et al. 1999; Papatheodorou et al. 2008; Fox et al. 2016). For instance, Fernandes and Monteiro (2017) examined the evolution in the number of authors of computer science publications, considering over 200,000 article references over a 60‐year period, from 1954 to 2014. Their study found a consistent increase in the average number of authors per paper across all decades. Moreover, Jang et al. (2016) investigated the increase in the number of authors per paper in Korean science and technology publications from 2000 to 2015, finding that the global trend of author growth per paper is evident in Korea as well.

However, there is limited literature addressing the increase in the number of authors when considering differences between countries. Indeed, while other aspects such as citations, self‐citations (Baccini and Petrovich 2023) and the number of publications has been investigated globally (Baccini and Petrovich 2023) and reported on platforms like SCIMAGO (https://www.scimagojr.com/), the global trend in the number of authors in neuroscience remains almost unexplored.

Understanding why the number of authors is increasing is important, as this trend may reflect broader dynamics in the academic and research field. For instance, increased authorship can signal greater collaboration, which is often required to tackle the growing complexity of experimental methodologies and techniques in scientific fields (Abt 2007). In neuroscience, for instance, research frequently relies on sophisticated technologies and large‐scale collaborations, such as neuroimaging studies, global brain mapping initiatives and developing biologically based models of cognitive abilities. We believe that these initiatives, which require diverse expertise and substantial resources, are likely significant contributors to the rising number of contributors per publication.

On the other hand, rising authorship numbers may also reflect systemic pressures, such as the ‘Publish or Perish’ culture and academic evaluation systems that prioritize metrics like h‐indices (Zerem 2017) and citation counts (Baccini and Petrovich 2023). These systems may inadvertently encourage practices like honorary authorship (Wislar et al. 2011), where individuals are included as co‐authors without significant contributions, raising ethical concerns.

A first step was taken in our previous study (Paul et al. 2024), where we analysed the rise in the number of authors in neuroscience publications within the G10 countries. Here, we extend our research by studying the global increase in the number of authors in the field, aiming to better understand the collaborative patterns in neuroscience across countries. We will also examine the relationship between the number of authors in publications and research fundings, international and between institution collaborations and number of publications.

## 2 Methods

We adopted a methodology analogous to our previous study on the increasing number of authors in neuroscience (Paul et al. 2024). The dataset for this analysis comprised all neuroscience publications from 2001 to 2022, sourced from 306 neuroscience journals indexed in the Web of Science (WoS, www.webofscience.com).

In this study, we included only countries with a minimum of 450 total publications over the 2001–2022 period. This threshold was selected for two reasons. First, we posited that a smaller number of articles would not provide a reliable average number of authors per article, as a limited dataset is more susceptible to the influence of outliers. Second, many countries with fewer than 450 articles displayed years with zero publications. By applying this threshold, we analysed 49 countries, encompassing a total of 843,231 publications. The results from some of the countries have been already published in our previous article (Paul et al. 2024) and are included for comparison.

Moreover, the number of authors per publication was anomalously high in some cases, with some publications exceeding 400 authors. Possible explanations for this high number have been already discussed in Paul et al. (2024). Following the methodology of our previous article (Paul et al. 2024), we excluded publications with more than 40 authors.

### 2.1 Data Analysis

As in our previous study on this topic, we used a MATLAB script generated in that previous work to identify the country name from the address of the corresponding author. The number of authors was determined by counting the semicolons (;) in the author list and adding one, as the last author is followed by a dot and not by a semicolon.

We then constructed a matrix containing the number of authors per paper, the country of the corresponding author and the year of publication. These data were used to calculate the average number of authors per year from 2001 to 2022. To assess changes in authorship patterns over time, we employed the delta measure, comparing differences between two subperiods (2001–2011 and 2012–2022).

To explore potential factors associated with the number of authors, we analysed metrics related to international collaboration, institutional affiliations and number of publications. The number of international collaborations was determined by examining the addresses associated with the authors, identifying distinct country names from a predefined list of recognized nations and counting the number of unique countries involved. If all affiliations were from the same country, the count of international collaborations was set to zero. Similarly, the number of institutional affiliations was calculated by extracting the affiliation strings provided for each author, splitting them into individual entries using semicolon delimiters, removing duplicates and counting the unique institutions.

To investigate potential factors associated with authorship trends, we also correlated the average number of authors per publication with national research funding levels. Data on research funding were sourced from the World Bank's DataBank, specifically the ‘Research and development expenditure (% of GDP)’ indicator.

To maintain methodological consistency, all correlation analyses were restricted to data spanning 2001 to 2021. Data from 2022 were excluded because of the lack of World Bank's available funding information for that year. For the same reason, Taiwan was omitted from the correlation analyses, as funding data were not available.

To statistically test whether the number of authors differed significantly between countries and across periods, we performed a two‐way mixed ANOVA (49 × 2), with Country as the between‐subjects factor and Sub‐period as the within‐subjects factor. Additionally, Pearson correlation analyses were conducted to examine the relationships between the number of authors per publication and factors such as international collaborations, institutional affiliations, number of publications and national research funding levels. All statistical analyses were performed using both MATLAB R2021 (www.mathworks.com) and SPSS (https://www.ibm.com/spss).

## 3 Results

**FIGURE 1:**
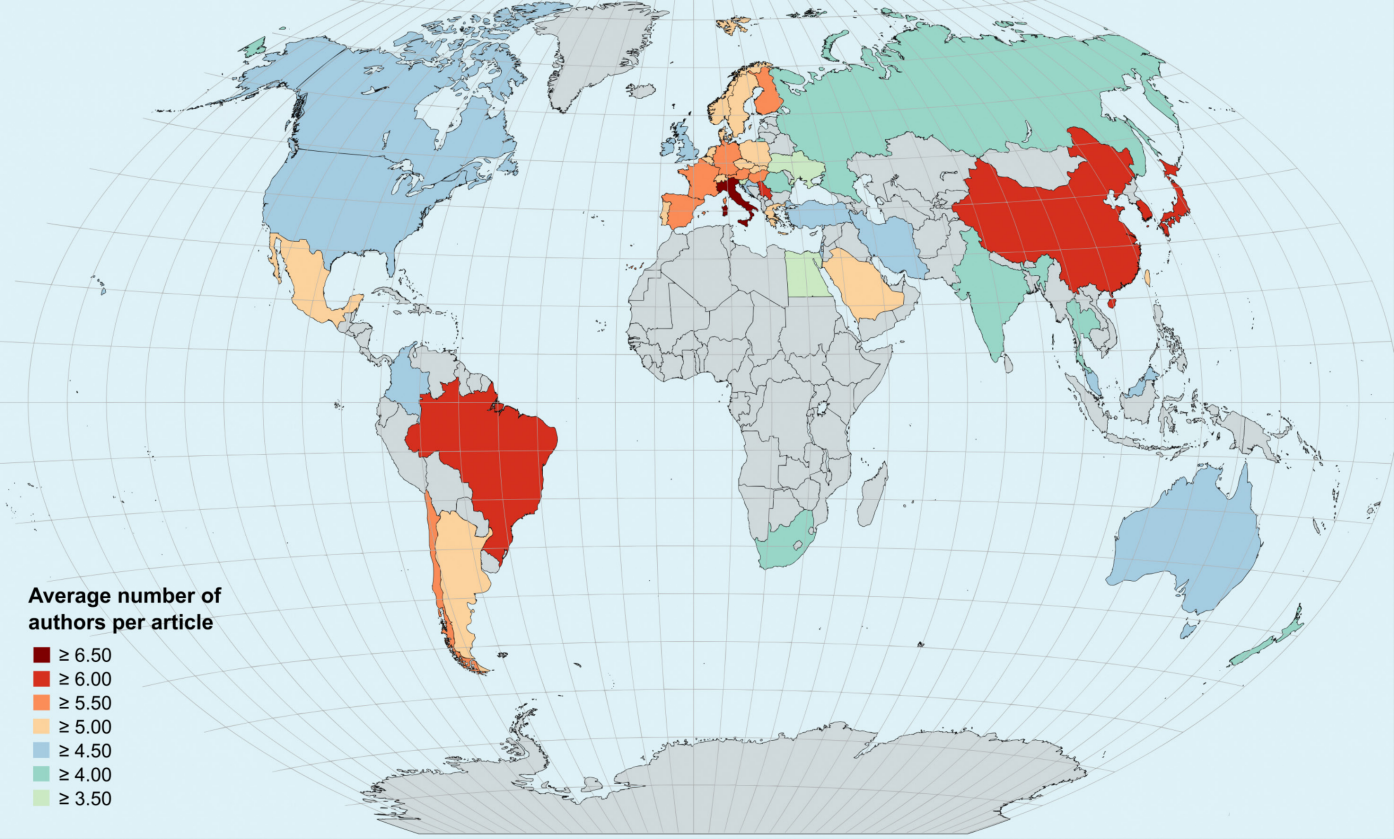
The average number of authors per country in the field of neuroscience. Only countries with a minimum of 450 published articles were included in this analysis, while other countries are coloured in grey. The map was created using MapChart (https://www.mapchart.net/).

Figure 1 provides a global overview of the average number of authors per country in neuroscience.

The figure shows that Italy has the highest average number of authors, with 6.5 authors per article, followed by South Korea, China, Serbia, Japan and Brazil, each with more than six authors per article.

While Canada is the G10 country with the lowest number of authors, as shown by Paul et al. (2024), we observed that other countries, including nations like Egypt and Ukraine, have even lower numbers.

**FIGURE 2:**
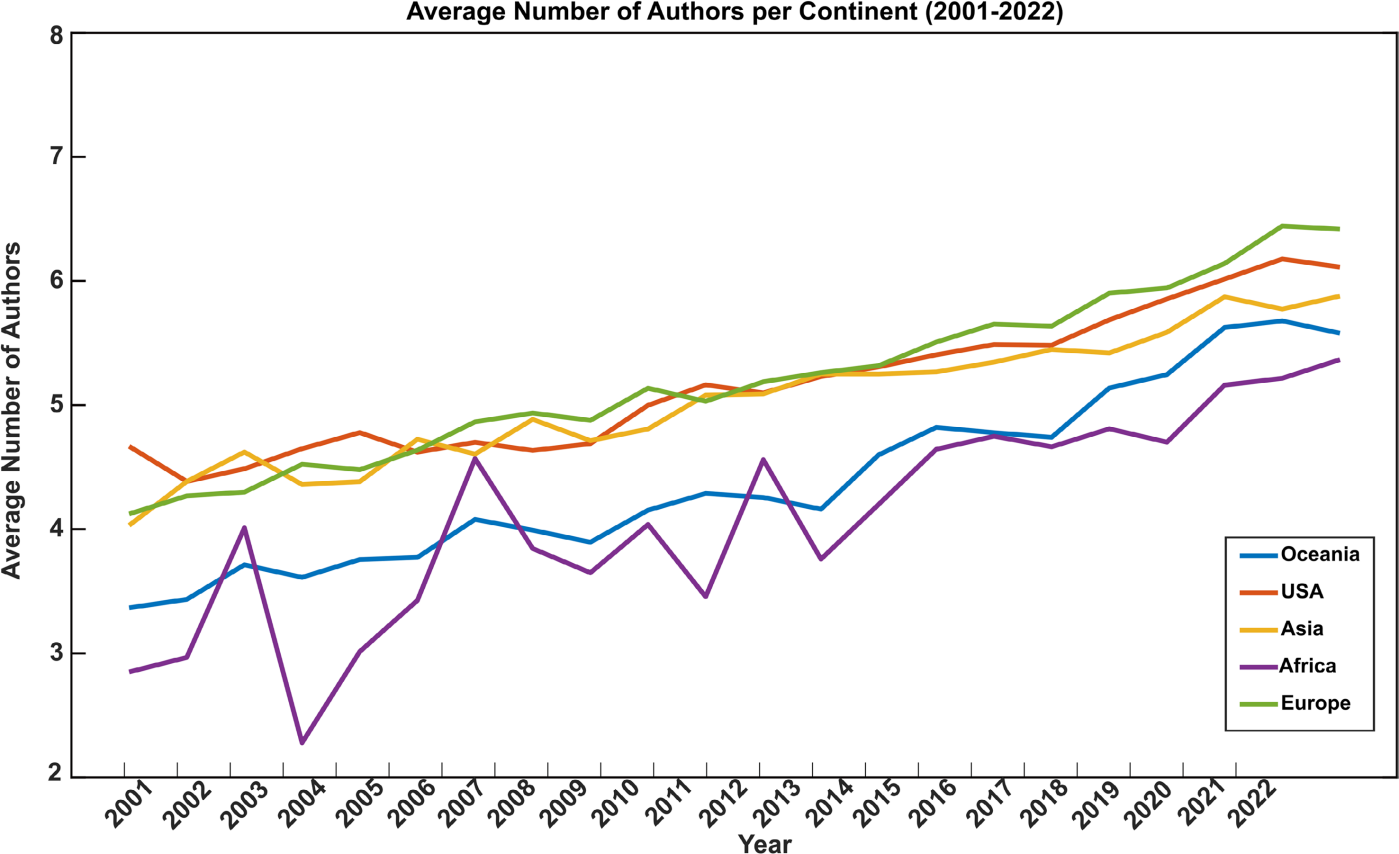
Trend of the average number of authors per article from 2001 to 2022 across different continents (Oceania, USA, Asia, Africa and Europe). The lines represent the average number of authors for each continent, including only data from the 49 analysed nations.

To contextualize these national differences within a global framework, we examined the continental trends in the number of authors over the studied decades. Figure 2 presents this trend for each continent (Africa, Oceania, Asia, America and Europe); this figure is only for illustrative purposes as the detailed explanation and statistical analysis are performed later. Figures S1–S5 provide detailed data for each of the 49 countries studied.

**FIGURE 3:**
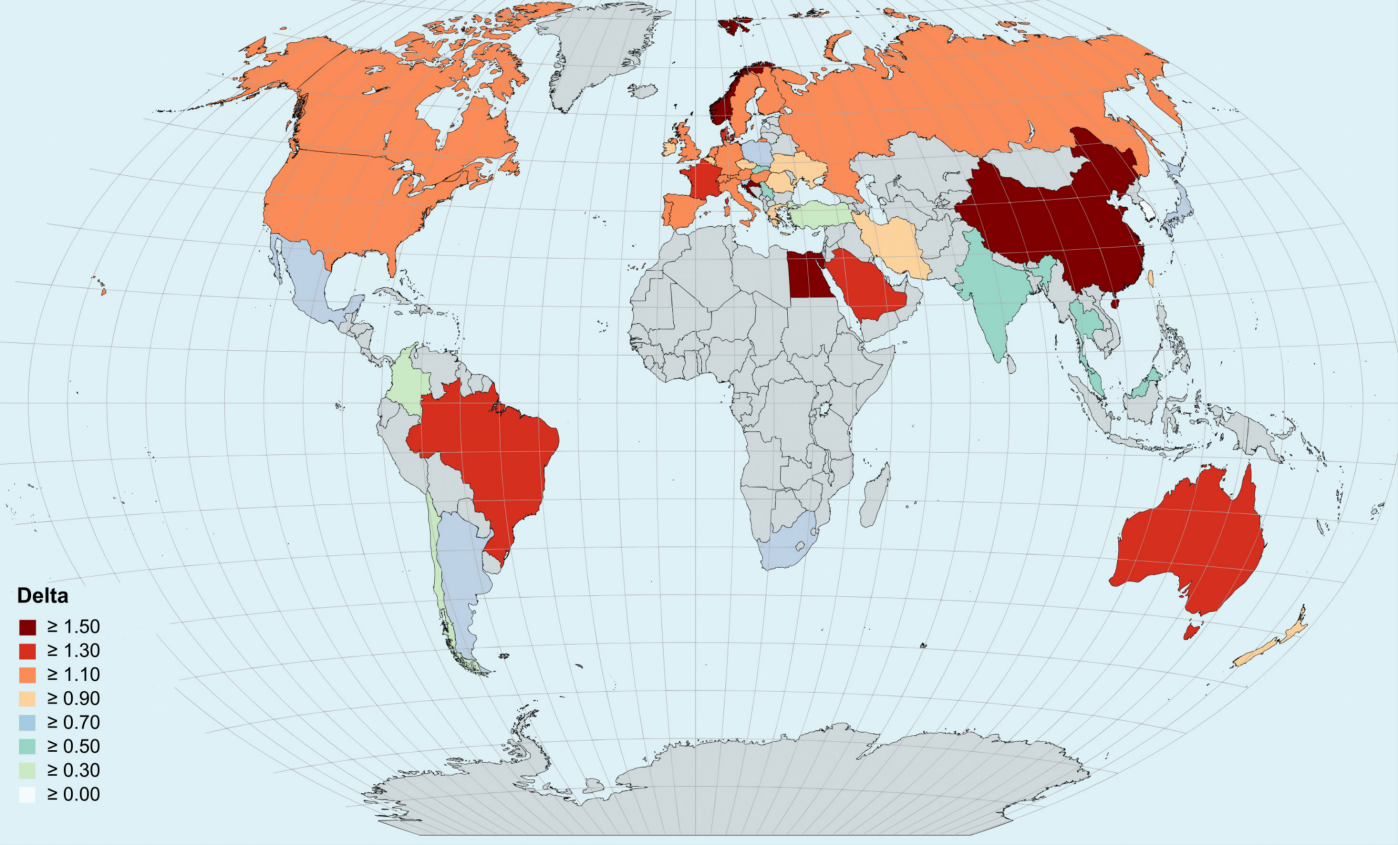
Map displaying the delta values in the field of neuroscience, representing the change between these two subperiods (2001–2011; 2012–2022), for each country. Only countries with a minimum of 450 published articles were included in this analysis, whereas the other countries are coloured in grey. The map was created using MapChart (https://www.mapchart.net/).

The ranking of the 49 countries is reported in detail in the Supporting Information (Table S1), which also reports a quantification of the growing number of authors per publication calculated as a delta between the 2012–2022 and 2001–2011 decades. The magnitude of this delta for each county is graphically represented in Figure 3.

A two‐way mixed ANOVA revealed that the number of publications was significantly influenced by both the Country (*F*(48,490) = 21.24, *p* < 0.001) and Sub‐period factor (*F*(1,490) = 1328.2, *p* < 0.001), which highlights a significant increase of the average number of authors in the 2011–2022 decade. Additionally, the interaction between the two factors was statistically significant (*F*(48, 490) = 3.21, *p* < 0.001). China shows the largest delta between the two subperiods, followed by Norway and Egypt. South Korea is the only country showing a slight decreasing trend rather than growth (Figure 2).

Newman–Keuls post‐hoc comparisons between countries indicate that the number of authors of the first‐ranking countries (Italy, South Korea, China, Serbia, Japan and Brazil) was significantly higher than the rest of the countries (all *p*s < 0.05), while it did not differ between them (all *p*s > 0.05). Similarly, the number of authors of the lowest ranking (Romania, Thailand, New Zealand, Russia, India, Slovenia, Egypt and Ukraine) differed significantly from the rest of the countries (all *p*s < 0.05), while their values were comparable (all *p*s > 0.05). Country‐by‐country Newman–Keuls post‐hoc comparisons are presented in Figure S6.

### 3.1 Factors Associated With the Increase in Number of Authors

**FIGURE 4:**
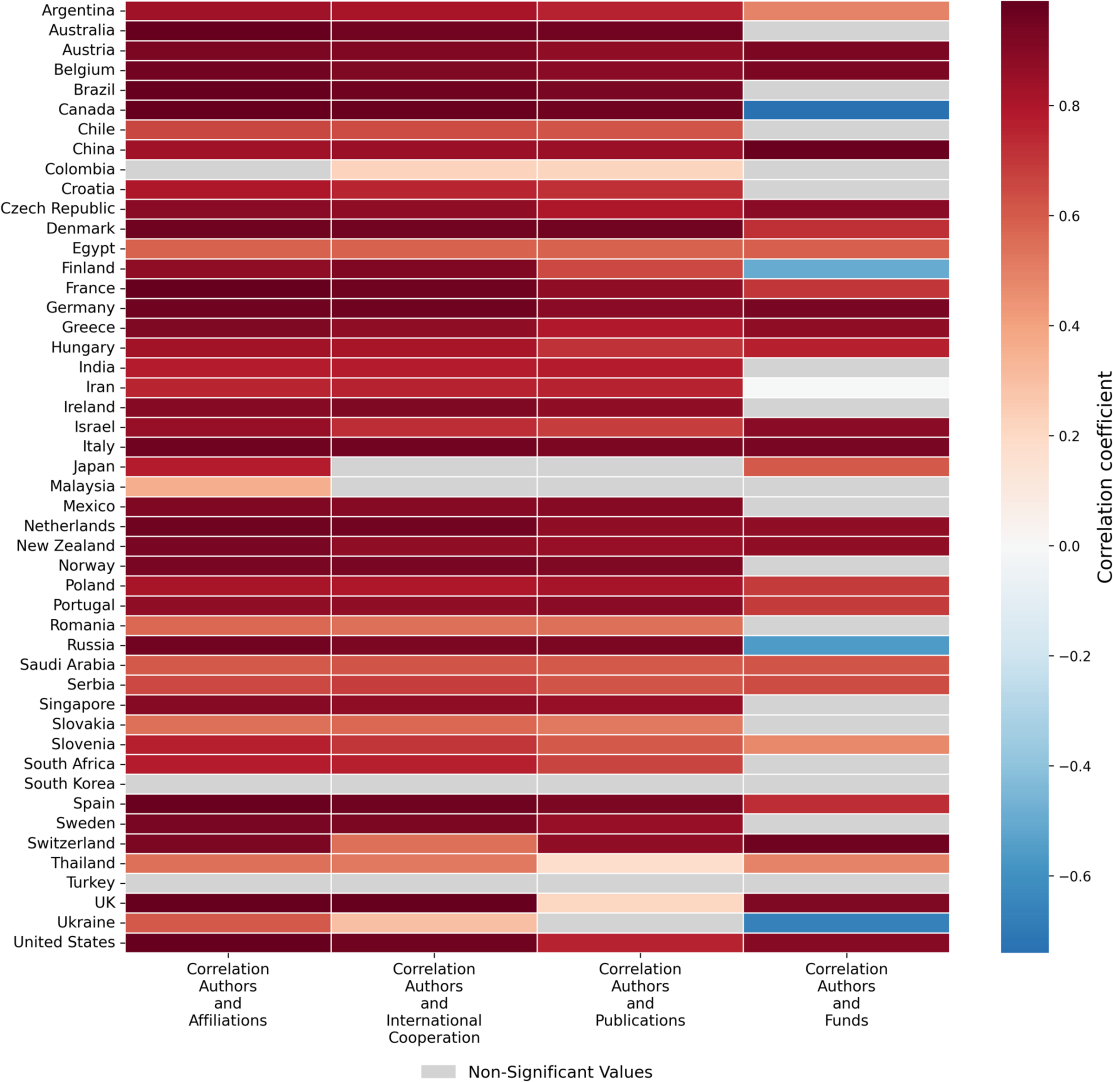
Heatmap of correlation coefficients for factors associated with the number of authors per neuroscience publication. Pearson correlation coefficient (*r*) quantifies the strength and direction of associations between the number of authors per publication and various contributing factors across countries. Nonsignificant correlations (*p* ≥ 0.05) are shown in grey.

In addition to our primary analyses on the number of authors per neuroscience publication, we conducted further correlations to explore factors potentially contributing to this increase. The Pearson correlation coefficient was used to measure the strength and direction of these relationships (Figure 4); the full table of correlation coefficients is included in the Supporting Information (Table S2).

These analyses aimed to identify patterns that might travel alongside the growth in authorship numbers, providing insights into possible drivers of this phenomenon. The results revealed a strong positive correlation between the number of authors and both international collaborations and institutional affiliations in most countries. For instance, in the United Kingdom, the correlation between authorship and international collaboration was particularly high (*r* = 0.99), and similarly strong correlations were also observed for institutional affiliations (*r* = 0.98). Similarly, in China, both international collaborations (*r* = 0.85) and institutional affiliations (*r* = 0.84) exhibited strong correlations with the number of authors. However, the correlations were not uniform across all countries. For example, South Korea showed nonsignificant correlation between authorship and international collaborations, and with institutional affiliations as well.

Furthermore, to investigate whether research funding levels are associated with the increase in the number of authors per neuroscience publication, we correlated the average number of authors per paper with available data on national research funding for each country. This analysis aimed to identify whether funding availability influences collaborative behaviour or other factors that contribute to authorship trends.

In our analysis, data on research funding were sourced from the World Bank's DataBank, specifically using the indicator ‘Research and development expenditure (% of GDP)’. This dataset provides a standardized measure of national investments in research and development as a percentage of gross domestic product (GDP) for each country. The data are currently publicly available at World Bank DataBank, covering the period up to 2021. Data for Taiwan were not available in the World Bank dataset, but the remaining countries were well represented.

The results showed mixed patterns. In several countries, a strong positive correlation was observed, suggesting that higher funding levels are associated with an increase in the number of authors per publication. For example, in China (*r* = 0.97) and Italy (*r* = 0.94), the correlation coefficients suggest that increased funding supports larger, more collaborative research efforts. Conversely, in some countries, we observed negative correlations. For instance, Ukraine (*r* = −0.67) showed inverse relationships. Interestingly, several countries exhibited weak or negligible correlations, such as South Korea (*r* = 0.041).

Finally, our analysis explored the relationship between the number of authors and the total number of publications in neuroscience across various countries. The findings revealed a consistent trend: in many countries, we observed a strong positive correlation between the two variables, indicating that an increase in the number of authors per publication is often associated with a higher volume of scientific output. For instance, in Brazil, a strong correlation was observed (*r* = 0.94). However, also in this case, the relationship was not consistent across all countries. For example, once again, South Korea showed a negligible and nonsignificant correlation (*r* = 0.1), indicating that the number of authors per paper had little association with the overall publication output.

## 4 Discussion

Examining the increase of authors worldwide, we found that the increasing trend of authorship in the field of neuroscience is a global phenomenon. This pattern persists even in countries where the number of publications is relatively low, although the analysis was restricted to countries with at least 450 articles. However, the number of articles is also gradually increasing in countries that are currently underrepresented, such as Africa and South America. This is evidenced by data from the SCIMAGO site (https://www.scimagojr.com/), which displays the number of publications on Scopus for each country from 1996 to 2023.

Italy stands out as the country with the highest average number of authors per article in the field of neuroscience. On the other hand, regarding the largest growth from the first subperiod (2001–2011) to the second (2012–2022), China exhibited the highest increase in authorship, followed by Norway and Egypt.

The rapid growth in authorship in China can be partially attributed to the ‘Publish or Perish’ culture that is widely prevalent in the country (Tian et al. 2016). This pressure is particularly intense regarding publishing in international journals and collaborating with authors from other countries. Indeed, Fu et al. (2011) conducted a bibliometric evaluation of highly cited papers from 1999 to 2009 using the Essential Science Indicators (ESI) database across 22 scientific fields, including neuroscience. They found that 47% of all Chinese ESI papers were international collaborations involving 101 countries. Furthermore, Fu's analysis revealed that a significant number of the most cited papers were authored by numerous individuals, with some papers having over 200 authors from more than 8 countries. Therefore, the pressure for international publications and the extensive involvement of multiple countries could be significant factors contributing to the substantial growth in the number of authors in Chinese articles.

Our analysis also showed a strong correlation between the number of authors and international collaborations in China (*r* = 0.85). These findings reinforce the role of collaborative efforts beyond national borders, as a driver of the increasing authorship trend in China.

The increase in authorship in Egypt can possibly be explained, by taking a cue from a nonneuroscientific field. In fact, a study by Farahat (2002) examined authorship patterns in the agricultural sciences in Egypt by analysing all papers published in 19 scientific journals. The findings showed a dominant trend toward multiple authorships, with a steady increase in multiauthor papers from 69% in 1960 to 89% in 1980. According to Farahat, a reason for the increasing cooperation is the pressure to collaborate to share the publication costs. As these costs increase, there is a greater incentive for researchers to involve colleagues in sharing the expenses, often resulting in the addition of their names as co‐authors, irrespective of their actual contributions to the work.

This rationale presents a plausible explanation, and consequently, it can be anticipated that as the number of publications increases in other nations facing substantial economic challenges, the number of contributing authors will similarly rise. This perspective is particularly relevant for interpreting the trends observed in Ukraine, where the number of authors per publication increased despite a negative correlation between funding and authorship (*r* = −0.67). In Ukraine, limited funding availability may have driven researchers to adopt collaborative strategies, pooling resources and distributing financial or material burdens to sustain research productivity.

However, while economic constraints appear to play a similar role in fostering collaboration in both Egypt and Ukraine, the outcomes differ. Egypt demonstrated a positive correlation between funding and authorship (*r* = 0.59), suggesting that increased funding further supports collaborative efforts. This divergence underscores the complexity of the relationship between funding and authorship trends. It likely indicates that, while economic pressures often incentivize collaboration, the ways these pressures translate into authorship practices are shaped by additional factors, including academic norms, funding mechanisms and cultural expectations.

The economic explanation was overlooked in Paul et al. (2024) but should be expected to account, at least in part, for the increasing trend in authorship in other countries to meet the increasing financial costs associated with the publication process, especially since many journals have moved toward open access, shifting the cost of publication from the readers or university subscriptions to the authors.

However, this rationale carries less weight in economically prosperous countries such as Norway. Nevertheless, Nylenna et al. (2014) investigated practices among Norwegian researchers by administering a questionnaire to researchers and PhD students at Oslo University Hospital and the University of Oslo (2700 individuals). They found that 36% of respondents had experienced pressure to include authors in their papers who had not made significant contributions. This could be another of the reasons for the significant growth in the number of authors in such settings.

The relationship between the number of authors per publication and the total number of publications provides additional insights into these trends. In countries with a high volume of scientific output, such as Italy and China, the number of authors per article appears closely linked to the overall publication growth. This relationship suggests that larger collaborative efforts may drive higher productivity in terms of publication volume. In Italy, for instance, the rise in both authorship and publication numbers aligns with the country's stringent academic evaluation processes, which emphasize quantitative metrics like publications and citations as benchmarks for career advancement. Similarly, China's rapid growth in authorship is accompanied by an increase in total publications, driven in part by intense pressure to publish in international journals and collaborate globally.

Finally, the considerable growth in the number of authors per article in Italy can be accounted for, at least in part, by the inclusion of honorary authors as a response to the stringent academic evaluation processes employed within the country, particularly in the context of higher education and research institutions (de Santis Puzzonia et al. 2018). Indeed, in Italy, advancement to ranks such as associate or full professorship is contingent upon meeting specific benchmarks that include the number of publications, citations and h‐index values over a defined period. These criteria are part of a broader framework managed by the Agency for the Evaluation of the University and Research System (ANVUR), which is responsible for setting and recalibrating these thresholds across various academic fields and ranks. The impact of such evaluation criteria on publication practices is profound, because it incentivizes researchers to increase their publication output, often through collaborative efforts, to meet the required thresholds.

Therefore, the reasons behind the worldwide increase in the number of authors per article are not only linked to the growing complexity of scientific subjects (Weeks et al. 2004; Abt 2007) but also to factors related to the economies of countries, peer pressure and an academic system that relies predominantly on quantitative indicators. This convergence of factors highlights a significant ethical dilemma that has been thoroughly examined in our previous publication (Paul et al. 2024) and that we re‐examine only briefly here.

The ethical concern arises from the prevailing ‘Publish or Perish’ culture within academia, which employs publication metrics as critical criteria for career advancement. In countries like Italy, these metrics often can lead to a proliferation of multiauthorship where not all listed contributors have played a meaningful role in the research. This ethical issue highlights the need for normalization by the number of authors to assign a fair score to the individual scientist, as proposed for the H index (Schreiber 2008; Galam 2011; Zerem 2017). The strong impact of the ‘Publish or Perish’ culture is evident not only in the increasing number of authors but also in the rise of self‐citations in certain countries. In fact, Baccini and Petrovich (2023) compared self‐citation rates across 50 countries between 1996 and 2019. They found that, while self‐citations generally decreased over time in most countries, 12 countries (Colombia, Egypt, Indonesia, Iran, Italy, Malaysia, Pakistan, Romania, Russia, Saudi Arabia, Thailand and Ukraine) showed the opposite trend.

Our findings on the high number of authors in Italy and the rapidly increasing trend in Egypt align with the high number of self‐citations. In contrast, in our work, the other 10 countries identified by the study of Baccini and Petrovich (2023) are not ranked among the countries with the highest number of authors. This difference may suggest that researchers in different countries respond to diverse incentives and institutional pressures regarding citations and publication metrics.

Addressing the ethical complexities surrounding authorship, particularly issues like honorary and ghost authorship (Wislar et al. 2011), is essential for maintaining integrity in academic publishing. Honorary authorship, where individuals are included as co‐authors without significant contributions, and ghost authorship, where deserving contributors are excluded, raise significant ethical concerns. To mitigate these practices, many publishers have introduced policies requiring or formalizing the disclosure of author contributions (Vasilevsky et al. 2021). The introduction of guidelines, such as those by the International Committee of Medical Journal Editors (ICMJE, 2006), has provided a framework for defining authorship. Specifically, these guidelines aim to clarify roles, helping to prevent the inclusion of honorary authors or the exclusion of deserving contributors (Vasilevsky et al. 2021). However, at our knowledge, while guidelines like those from the ICMJE offer clear criteria and recommendations, their practical impact on authorship practices remains largely unexplored. Future research is needed to evaluate whether these policies have led to measurable improvements in fairness in authorship attribution.

To conclude, we believe that this work contributes to understanding these trends, which is crucial for informing research policies, promoting equitable collaboration and addressing ethical concerns such as honorary authorship and the potential overreliance on quantitative metrics in academic evaluations. While we identified factors related to the economies of countries, peer pressure, international collaboration and an academic system that relies on quantitative indicators, further studies addressing the system of incentives and country‐specific socio‐economic factors are needed to understand these differences. Moreover, while in this study we extended our initial research (Paul et al. 2024) to a global scale, we believe it is equally important to conduct the analysis using a country‐centred approach. This would involve examining the increase in the number of authors by geographic areas, specific universities, or even departments to understand the effect of local policies. We have limited our study to the field of neuroscience, but a more comprehensive study across all fields is needed, as some of our results may be specific to this field.

## Author Contributions

**Mariella Segreti:** conceptualization, data curation, formal analysis, writing – original draft, writing – review and editing, methodology, software. **Ann Paul:** conceptualization, data curation, formal analysis, writing – original draft, writing – review and editing, methodology, software. **Pierpaolo Pani:** conceptualization, writing – review and editing. **Aldo Genovesio:** conceptualization, formal analysis, supervision, validation, writing – original draft, writing – review and editing. **Emiliano Brunamonti:** conceptualization, formal analysis, supervision, validation, writing – original draft, writing – review and editing.

## Conflicts of Interest

The authors declare no conflicts of interest.

### Peer Review

The peer review history for this article is available at https://www.webofscience.com/api/gateway/wos/peer‐review/10.1111/ejn.70092.

## Supporting information


**Figure S1** Temporal trend of authorship in the field of Neuroscience for the African target countries from 2001 to 2022.
**Figure S2.** Temporal trend of authorship in the field of Neuroscience for the Oceanic target countries from 2001 to 2022.
**Figure S3.** Temporal trend of authorship in the field of Neuroscience for the Asian target countries from 2001 to 2022. We divided the Asian countries into two separate figures following the regional classifications defined by WorldAtlas (www.worldatlas.com/), in order to avoid excessive overlapping between countries. a. Average number of authors per year in Northern, South and Southeast Asia. b. Average number of authors per year in East Asia and Middle East.
**Figure S4.** Temporal trend of authorship in the field of Neuroscience for the American target countries from 2001 to 2022.
**Figure S5.** Temporal trend of authorship in the field of Neuroscience for the European target countries from 2001 to 2022. We divided the European countries into four separate figures to avoid excessive overlapping between countries. The division follows the regional classifications of Eastern, Western, Northern, and Southern Europe, as defined by the United Nations Geoscheme for Europe. a. Average number of authors per year in Eastern Europe. b. Average number of authors per year in Western Europe. c. Average number of authors per year in Northern Europe. d. Average number of authors per year in Southern Europe.
**Figure S6.** Country‐by‐Country Newman–Keuls post‐hoc comparisons.
**Table S1.** Average number of authors, along with the standard deviation, across all journals in the field of neuroscience for the period from 2001 to 2022.
**Table S2.** Correlation analyses exploring factors associated with the number of authors per publication.

## Data Availability

The entire dataset used for this analysis is available in: Paul, A. (2024). Neuroscience publications (2000–2022) [Data set]. Zenodo. 10.5281/zenodo.11445060.
